# Associations of severe hypophosphatemia and clinical outcomes in mechanically ventilated children with dengue shock syndrome

**DOI:** 10.1097/MD.0000000000045673

**Published:** 2025-11-07

**Authors:** Luan Thanh Vo, Thanh Tat Nguyen, Bao Trung Nguyen, Phuong Thi-Mai Ngo, Dung Thi-Thuy Pham, Thanh Thi-Hoai Mai, Thach Ngoc Pham, Tung Huu Trinh

**Affiliations:** a Department of Infectious Diseases, Children’s Hospital 2, Ho Chi Minh City, Vietnam; b Department of TB, Woolcock Institute of Medical Research, Ho Chi Minh City, Vietnam; c Family Medicine Department, University of Medicine and Pharmacy, Ho Chi Minh City, Vietnam; d Department of Pediatric Nephrology, Children’s Hospital 2, Ho Chi Minh City, Vietnam; e Department of Pediatric Cardiology, Children’s Hospital 2, Ho Chi Minh City, Vietnam.

**Keywords:** dengue shock syndrome, hypophosphatemia, mechanical ventilation, mortality, phosphorus imbalance, vasopressors

## Abstract

Hypophosphatemia is common in critically ill children, but its clinical implications in dengue shock syndrome (DSS) remain uncertain. This retrospective single-center study, conducted in 2022 at a tertiary pediatric intensive care unit in Vietnam, investigated the association between marked phosphorus reduction and clinical outcomes in children with DSS requiring mechanical ventilation (MV). Fifty-seven children with DSS on MV and complete phosphorus profiles were enrolled. Propensity score matching adjusted for key confounders including age, serum calcium, metabolic acidosis, and creatinine levels. The primary outcome was in-hospital mortality; secondary outcomes included the duration of MV and vasopressor use. Stepwise logistic and linear regression analyses were applied. The median patient age was 6 years (interquartile range [IQR]: 4–9). Twenty-one patients (37%) developed severe hypophosphatemia during pediatric intensive care unit admission. Overall mortality was 28% (16/57). Median MV duration was 5 days (IQR: 3–6), and vasopressor support lasted a median of 3 days (IQR: 1–5). While severe hypophosphatemia was not associated with mortality, it was significantly correlated with prolonged MV and vasopressor requirements. These findings suggest that severe hypophosphatemia may contribute to extended organ support. Timely recognition and correction of phosphorus deficits could provide potential clinical benefits in this vulnerable population.

**Key points**

- Children with dengue shock syndrome requiring mechanical ventilation (MV) have an in-hospital mortality rate of 28%.
- Severe hypophosphatemia was not associated with dengue shock syndrome-associated mortality rates.
- There were significant associations between severe hypophosphatemia and MV and vasopressor support length.
- Hypophosphatemia correction is recommended to reduce the risk of MV and vasopressor use in this critical cohort.

## 1. Introduction

Severe dengue is a major global health burden.^[1]^ Dengue shock syndrome (DSS) is a leading cause of pediatric intensive care unit (PICU) admissions in endemic regions, with a substantial proportion of children requiring mechanical ventilation owing to respiratory failure, prolonged shock, dengue-induced acute liver failure, and multiorgan dysfunction.^[2,3]^ Despite improvements in critical care management, mortality rates among mechanically ventilated children with DSS remain alarmingly high, ranging from 21% to 30%.^[4]^ Therefore, there is an urgent need to identify modifiable factors that lead to poor clinical outcomes, and appropriate interventions should be provided for this critical patient cohort.^[3,5]^

Among the metabolic disturbances in critically ill patients, hypophosphatemia is often overlooked, despite its significant clinical implications.^[6]^ Phosphorus is essential for ATP synthesis and plays a central role in energy metabolism and maintenance of vital organ functions. Severe hypophosphatemia, reported in nearly half of ICU patients, has been associated with adverse outcomes in critically ill populations.^[7–9]^ It has been shown that 69.2% of patients with severe dengue exhibited hypophosphatemia.^[10]^ Although hypophosphatemia has been extensively studied in the general ICU population, its role in PICU-admitted children with DSS remains poorly understood, with no existing established management including the 2009 World Health Organization (WHO) dengue guidelines.^[1]^

Several prognostic factors for poor outcomes in children with severe DSS have been reported, including prolonged shock, severe liver dysfunction, critical bleeding, coagulopathy, elevated lactate levels, and high cumulative fluid balance.^[2,3,5,11]^ However, the effect of severe hypophosphatemia on patients with DSS has not yet been explored. Hence, we hypothesized that a severe decrease in phosphorus levels might lead to adverse outcomes in mechanically ventilated children with DSS. This study aimed to investigate the association between severe hypophosphatemia and key clinical outcomes in patients with DSS on mechanical ventilation (MV), including in-hospital mortality, length of mechanical, and vasopressor use. Given the limited research in this area, our findings may provide valuable insights into the prognostic potential of phosphorus levels and help guide future research and management strategies for children with severe DSS in the PICU setting.

## 2. Methods

### 2.1. Study design, setting, and participants

This retrospective, single-center study was carried out at Children’s Hospital 2 in Ho Chi Minh City, Vietnam, a distinguished tertiary referral pediatric institution in southern Vietnam. We reviewed all cases of severe dengue hemorrhagic fever in patients admitted to the PICU in 2022. The eligibility criteria were age below 18 years, laboratory-confirmed dengue infection, and the presence of dengue shock syndrome requiring mechanical ventilation support. The exclusion criteria were a lack of serologically confirmed dengue infection and incomplete data on serum phosphorus levels.

### 2.2. Study outcomes

The primary outcome was in-hospital dengue-associated mortality, whereas secondary outcomes included the duration of mechanical ventilation support and vasopressor use.

### 2.3. Study definitions

Dengue infection was delineated in accordance with the WHO criteria from 2009, wherein confirmation was established through laboratory testing using the dengue-IgM antibody or nonstructural 1 antigen test.^[1]^ The diagnosis of dengue shock syndrome adhered to the guidelines outlined by the WHO for dengue in 2009.^[1]^ Severe hypophosphatemia was defined as phosphorus levels <0.48 mmol/L.^[12]^

Indications for mechanical ventilation in children with severe DSS were based on the Vietnamese Ministry of Health Dengue Guidelines.^[4]^ First, patients with severe DSS in whom the initial management of nasal continuous positive airway pressure fails are indicated for mechanical ventilation support. Further indications for MV included severe DSS manifesting as vast pleural and abdominal cavity effusions, abdominal compartment syndrome, pulmonary edema and/or fluid overload, acute respiratory distress syndrome, and high volume of continuous intravenous infusion (≥7 mL/kg/h) in numerous consecutive hours of infusion.^[4]^

### 2.4. Data measurements

Repeated measurements of serum phosphate levels during the PICU stay were performed using a machine-Alinity ci-series (Abbott, USA). Patient hemodynamics were assessed using the vasoactive inotropic score (VIS).^[13]^

### 2.5. Data collection

We enumerated a list of patients with DSS and mechanical ventilation admitted to the PICU in 2022. A total of 57 cases met the eligibility criteria, included in the study. Paper-based case report forms were used to collect clinical and laboratory data from the medical records at the time of admission, during the PICU stay, and at discharge. Data were meticulously entered into an electronic database for statistical analysis. The clinical outcomes of patients were assessed throughout their PICU stay and discharge.

### 2.6. Statistical analysis

Continuous variables are described using median and interquartile range (IQRs), whereas categorical variables are presented as numerical counts (n) and percentages (%). Missing data on serum phosphorus levels from retrospective data collection was the primary source of study bias. Only participants with complete phosphorus data were included in this analysis. Potential confounders, including age, pH, Ca^2+^ concentration, and creatinine level, can influence phosphorus levels in patients. Therefore, we implemented propensity score matching (PSM) for the predetermined baseline confounders.^[14,15]^ We performed backward stepwise model selection based on the akaike information criteria for all predetermined covariates using the PSM dataset. Statistical significance was set at *P*-values < .05 for all comparisons. The R statistical software (version 4.3.1, Boston, MA) was used for all analyses.

### 2.7. Ethics statement

Approval for this study was granted by the Institutional Review Board of Children’s Hospital 2, Ho Chi Minh City, Vietnam, with designated approval number 391, signed on March 24, 2022. To uphold adherence to Good Clinical Practice, all patient data were de-identified in the statistical analysis. The study was conducted in strict adherence to the principles of Good Clinical Practice and in accordance with the ethical guidelines of the Declaration of Helsinki.

## 3. Results

### 3.1. Baseline characteristics and clinical outcomes of study participants

A total of 57 mechanically ventilated children with DSS were enrolled and analyzed. Clinical and laboratory data of the study participants on admission are presented in Table 1. The median patient age was 6 (IQR: 4–9) years, and females accounted for approximately 53% of the participants. Twelve participants (21%) had underlying diseases. Among them, 67% had compensated DSS, and 33% had decompensated DSS. Critical bleeding was observed in 5 patients (9%). Upon admission, the median respiratory rate was 30 breaths/min (IQR, 23–30) breaths per minute. The median systolic shock index was 0.71 (IQR, 0.51–0.81) bpm/mm Hg and diastolic shock index was 0.44 (IQR, 0.37–0.56) bpm/mm Hg. Thirteen patients (22.8%) had high VIS (>30). Full blood counts revealed a marked reduction in platelet count. Severe hepatic transaminases (aspartate aminotransferase or alanine aminotransferase ≥ 1000 IU/L) were observed in 17 (30%) patients with DSS. The median serum creatinine level was 52 (IQR, 42–56) µmol/L. The median length of PICU stay was 7 (IQR, 6–8) days, and the median length of hospital stay was 12 (IQR, 9–15) days. The in-hospital mortality rate was 28% (of 16/57 patients died). The median length of mechanical ventilation support was 5 (IQR: 3–6) days, and that of vasopressor use was 3 (IQR: 1–5) days.

**Table 1:** Clinical and laboratory characteristics of study participants on PICU admission and associated clinical outcomes at discharge (N = 57).

Characteristics	Summary statistics
On PICU admission	
Age (yr)	6 (4–9)
Male sex, n (%)	30 (53)
Underlying diseases, n (%)	12 (21)
Grading of dengue severity, n (%)	
Compensated DSS	38 (67)
Decompensated DSS	19 (33)
Critical bleeding, n (%)	5 (9)
Glassgow coma scale (score)	15 (10–15)
Respiratory rate (breaths/min)	30 (23–30)
Systolic shock index (bpm/mm Hg)	0.71 (0.51–0.81)
Diastolic shock index (bpm/mm Hg)	0.44 (0.37–0.56)
White blood cell count (×10^9^/L)	8.0 (5.0–10.9)
Hemoglobin (g/dL)	12.8 (10.9–14.7)
Peak hematocrit, n (%)	42 (36–47)
Nadir hematocrit, n (%)	33 (29–39)
Platelet cell count (×10^9^/L)	25 (13–53)
Aspartate aminotransferase (AST), (IU/L)	604 (133–2156)
Alanine aminotransferase (ALT), (IU/L)	276 (49–724)
Severe transaminitis, n (%)	17 (30)
International normalized ratio	2.11 (1.42–2.84)
Blood lactate (mmol/L)	3.1 (2.1–5.6)
Serum creatinine (mmol/L)	52 (42–65)
Serum phosphorous levels (mmol/L)	
Baseline level upon admission	1.26 (1.02–1.46)
At 48 h since admission	0.87 (0.79–0.98)
Nadir level after 72 h post-admission	0.56 (0.34–0.75)
Arterial blood gas analysis	
pH	7.37 (7.30–7.41)
PCO_2_ (mm Hg)	24 (21–30)
PO_2_ with oxygen support (mm Hg)	124 (81–160)
Bicarbonate (mEq/L)	15.4 (12.6–17.7)
Cumulative fluid infused from referral hospitals and 24 h PICU admission (mL/kg)	276 (197–391)
Vasoactive inotropic score >20, n (%)	13 (22.8)
Outcomes of patients at discharge	
Length of hospital stay (d)	12 (9–15)
Length of PICU stay (d)	7 (6–8)
Days of mechanical ventilation	5 (3–6)
Days of vasopressor use	3 (1–5)
Fatal outcome, n (%)	16 (28)

Summary statistics are presented as median (interquartile range, IQR) for continuous variables and frequency (%) for categorical variables.

DSS = dengue shock syndrome, PICU = pediatric intensive care unit.

### 3.2. The correlations of baseline phosphorous level upon admission, the nadir value of serum phosphorous during PICU stay and clinical outcomes

**Figure 1.**
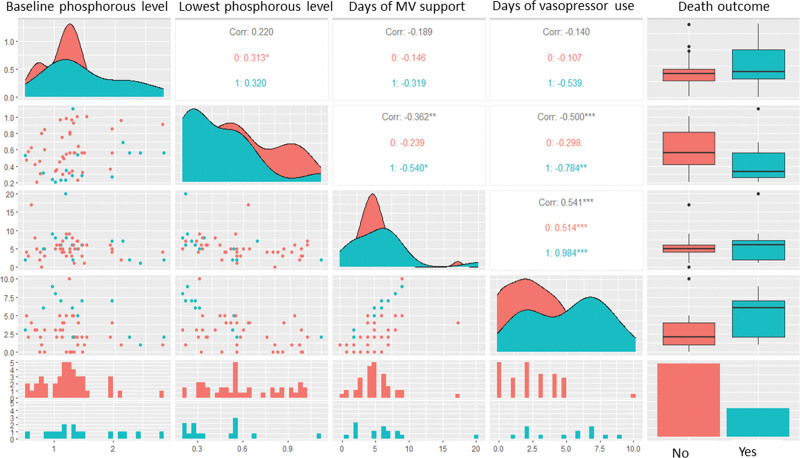
Correlation map of association between serum phosphorous levels and clinical outcomes in mechanically ventilated children with dengue shock syndrome.

The median of serum phosphorus concentration at admission was 1.26 mmol/L (IQR: 1.02–1.46), gradually declining with the median values of 0.87 (IQR: 0.79–0.98) mmol/L at 48 hours post-admission (Table 1). The nadir level of serum phosphorus among studied patients frequently occurred at 48–72 hours post-admission with the median value of 0.56 (IQR: 0.34–0.75) mmol/L. As shown in Figure 1, there were modest correlations between baseline phosphatemia and the duration (in days) of MV and vasopressor support. However, there were significant correlations between the nadir level of phosphatemia and duration of MV and vasopressor use. The associations were more pronounced in nonsurvivors (correlation coefficients of −0.54 for MV and −0.784 for vasopressor use) than in survivors (correlation coefficients of −0.239 for MV and −0.298 for vasopressor support). This reflects a greater reduction in serum phosphorus levels in nonsurvivors than in survivors.

### 3.3. Imbalance adjustments for baseline confounders by propensity score matching

**Figure 2.**
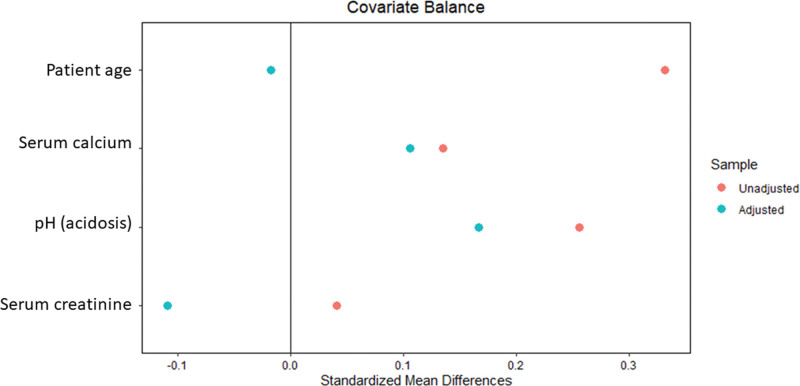
Propensity score matching (PSM) was performed for baseline confounders, including patient age, serum calcium level, acidosis, and serum creatinine level. After PSM, there was a marked improvement in the standardized mean differences in the group with adjusted confounders compared with the non-PSM group.

There was a marked imbalance (indicated by red dots in Fig. 2) in baseline confounders, including age, serum calcium, acidosis (pH), and serum creatinine, which interfered with the phosphorous levels of the study groups and clinical outcomes of interest. After PSM adjustment (denoted by blue dots in Fig. 2), the balance of confounders markedly improved in the regression analyses. The mean standardized propensity scores of all confounding variables significantly improved in the PSM cohort.

### 3.4. Association between nadir serum phosphorous and in-hospital mortality

As shown in Table 2, there was no significant association between nadir phosphatemia and mortality among mechanically ventilated patients with DSS in the bivariate and multivariate logistic regression analyses adjusted for severe bleeding, blood lactate level, vasoactive inotropic score, and sepsis.

**Table 2:** Associations between severe serum phosphorous reduction and in-hospital mortality among mechanically ventilated children with dengue shock syndrome.

Methods	Crude OR (95% CI)*	*P* *	Adjusted OR (95% CI)†	*P* †
Without PSM	3.11 (0.94–10.3)	.06	4.36 (0.73–26.1)	.10
With PSM	3.04 (0.93–9.92)	.07	4.47 (0.76–26.2)	.09

CI = confidence interval, OR = odds ratio, PSM = propensity score matching.

* Crude OR and *P*-values from bivariate analysis.

† The OR and *P*-value from multivariable logistic analysis, adjusted by severe bleeding, blood lactate, vasoactive inotropic score, and sepsis.

### 3.5. Association between nadir serum phosphorous and days of mechanical ventilation

As presented in Table 3, in the non-PSM cohort, both univariate and multivariate analyses showed a statistically significant association between severe hypophosphatemia and duration of mechanical ventilation in children with DSS (all *P* < .05). In the PSM cohort, the associations were adjusted slightly, with more accurate estimates and significant *P*-values.

**Table 3:** Linear regressions between severe serum phosphorous reduction and days of mechanical ventilation among mechanically ventilated children with dengue shock syndrome.

Models	Estimate	SE	*P*-value
Without propensity score matching			
Unadjusted model*	3.07	0.83	<.001
Adjusted model†	2.93	0.84	.001
With propensity score matching			
Unadjusted model*	2.75	1.07	.014
Adjusted model†	2.74	1.04	.012

Statistics are estimates of coefficients and standard errors (SE).

* Univariable linear regression analysis.

† Multivariable linear regression adjusted for severe bleeding, cumulative fluid infusion, sepsis, and phosphorous supplementation treatment.

### 3.6. Association between nadir serum phosphorous and duration of vasopressor use

As indicated in Table 4, the univariate and multivariable linear regressions (adjusted for covariates including severe bleeding, cumulative fluid infusion, sepsis, and phosphorus supplementation treatment) showed that the nadir levels of patients’ serum phosphorus during the PICU stay were highly associated with a longer duration of vasopressor use in the non-PSM and PSM models (all presented with significant *P* < .05).

**Table 4:** Linear regressions between severe serum phosphorous reduction and days of vasopressor use among mechanically ventilated children with dengue shock syndrome.

Models	Estimate	SE	*P*-value
Without propensity score matching			
Unadjusted model*	2.32	0.58	<.001
Adjusted model†	1.96	0.6	.002
With propensity score matching			
Unadjusted model*	2.1	0.73	<.01
Adjusted model†	1.96	0.79	.018

Statistics are estimates of coefficients and standard error (SE).

* Univariable linear regression analysis.

† Multivariable linear regression adjusted for severe bleeding, cumulative fluid infusion, sepsis, and phosphorous supplementation treatment.

## 4. Discussion

In this single-center retrospective study, we examined the correlations between severe hypophosphatemia and clinical outcomes, including the duration of mechanical ventilation, vasopressor use, and in-hospital mortality, among pediatric patients with severe DSS requiring mechanical ventilation in the PICU. This study showed that children with severe hypophosphatemia are more likely to experience worse clinical trajectories. Although the association between severe hypophosphatemia and mortality did not reach statistical significance, nadir phosphorus levels were significantly associated with prolonged mechanical ventilation and extended vasopressor use even after adjusting for potential confounders.

To date, the effects of hypophosphatemia on in-hospital mortality, duration of mechanical ventilation, and vasopressor use in children with severe DSS requiring intensive care have not been fully explored. Several factors may have contributed to severe hypophosphatemia in this critical study population. First, prolonged shock, commonly observed in children with severe DSS requiring mechanical ventilation, can lead to reduced intestinal phosphorus absorption due to ischemia of the gastrointestinal tract.^[4,11]^ Second, parenteral nutrition, which was observed in 68.4% of cases, promotes intracellular phosphorus transport and exacerbating hypophosphatemia.^[16]^ Additionally, sepsis, acute kidney injury, and excessive systemic inflammatory responses, which are frequently observed in severe DSS, may further worsen hypophosphatemia through phosphorus redistribution.^[4,17,18]^ Moreover, prolonged use of catecholamine vasopressors has been reported to aggravate hypophosphatemia, compounding its severity.^[19]^ Therefore, we performed propensity score matching to minimize the confounding bias effect, which potentially influences the baseline differences between children with and without severe hypophosphatemia. Thus, PSM dramatically enhanced the reliability and validity of our study. To the best of our knowledge, there is currently insufficient data to evaluate the impact of hypophosphatemia on mortality in children with DSS and MV. To our knowledge, no previous studies have evaluated the effect of hypophosphatemia on mortality in children with DSS. Notably, we recently reported that 13% of PICU-admitted children with severe DSS require mechanical ventilation support, with mortality rates ranging from 21% to 30%, despite intensive treatment.^[2,4,11]^ Hence, we conducted this exploratory study and found no significant correlation between severe hypophosphatemia and DSS-related mortality. Our findings are inconsistent with those of previous reports on other critically ill patients.^[7–9]^ Possible explanations include the distinct characteristics of patients with severe DSS on MV, and the limited sample size required to detect significant differences in mortality in this study cohort.

Phosphorus is essential for ATP synthesis and provides energy to respiratory muscles. Therefore, phosphorus deficiency leads to muscle weakness, respiratory fatigue, and difficulty weaning from mechanical ventilation. Our study demonstrated a correlation between severe hypophosphatemia and prolonged mechanical ventilation, with each 1 mmol/L reduction in the lowest serum phosphorus levels associated with an increase of approximately 3 days of ventilator dependence. This finding is consistent with those of previous studies.^[8,20,21]^ A plausible explanation for this prolonged ventilation is that phosphorus is a key factor in ATP production, which is required in large quantities by the respiratory muscles, including the diaphragm, thereby increasing the duration of mechanical ventilation.^[21]^ Correction of phosphorus levels has been reported to benefit ventilator weaning, suggesting the potential value of early intervention for severe hypophosphatemia in mechanically ventilated patients.^[22]^ Additionally, hypophosphatemia can lead to arrhythmias, reduced myocardial contractility, and increased vasopressor requirements, owing to impaired energy metabolism in cardiac cells.^[16]^ Phosphorus supplementation has been shown to improve the ejection fraction by up to 20%.^[22]^ Furthermore, hypophosphatemia has been associated with neurological dysfunction, including acute encephalopathy, seizures, apnea, and delirium, all of which may prolong mechanical ventilation.^[23]^

Notably, severe bleeding and a high VIS (>30) have been demonstrated to increase the risk of mortality in patients with dengue shock syndrome.^[3,5,24]^ Severe hypophosphatemia can potentially impair platelet hemostatic function, leading to thrombocytopenia and contributing to hemorrhagic complications such as uncontrolled bleeding, acute blood loss, prolonged shock, and reduced organ perfusion in patients with DSS.^[25,26]^ Furthermore, severe hypophosphatemia may contribute to endothelial dysfunction, which can disrupt vascular homeostasis.^[27]^ Furthermore, this disruption can exacerbate vascular hyperpermeability, resulting in increased and prolonged plasma leakage, further complicating the clinical management of patients with DSS. Our study identified an association between severe hypophosphatemia and extended vasopressor use in children with severe DSS, with each 1 mmol/L decrease in the lowest phosphorus levels associated with an increase of approximately 2 additional days of vasopressor support compared to those without severe hypophosphatemia.

This study provides a comprehensive evaluation of the impact of severe hypophosphatemia in critically ill children with DSS, particularly in those requiring mechanical ventilation. Although no significant association was observed between hypophosphatemia and mortality, our findings have important clinical implications in respiratory and cardiovascular support. Severe hypophosphatemia was significantly associated with extended duration of mechanical ventilation and vasopressor use. These associations are likely driven by the critical role of phosphorus in cellular energy production, which is essential for respiratory muscle function, myocardial performance, and vascular stability. Therefore, phosphorus deficiency may lead to respiratory fatigue, impaired cardiac output, and increased vascular permeability, all of which can complicate recovery in DSS patients. This study also benefitted from the application of propensity score matching, which enhanced the validity of the findings by minimizing confounding bias. Overall, this underscores the need for the timely detection and management of hypophosphatemia to improve clinical outcomes in critically ill children with DSS.

Although phosphorus correction has been proposed as a means of shortening the duration of vasopressor use and mechanical ventilation, strong supporting evidence remains inadequate. This retrospective study has several limitations, particularly the small sample size and insufficient statistical power, which may have limited the applicability of the results, increased the risk of sampling bias, and weakened the overall reliability of the conclusions. Additional limitations include the single-center design, retrospective nature of the cohort, inconsistent collection of clinical and laboratory data, and presence of unforeseen confounding factors that could influence phosphorus levels. Nevertheless, we addressed potential confounding factors by applying propensity score matching to balance baseline characteristics between the groups. Although this study offers valuable insights, the findings should be interpreted with caution and further studies involving larger and more diverse populations are necessary to confirm these results.

## 5. Conclusions

This study demonstrated an association among hypophosphatemia, prolonged mechanical ventilation, and vasopressor use in children with severe DSS. Monitoring phosphorus levels and hypophosphatemia correction are of utmost clinical significance, regarding the clinical benefits of shortening the duration of mechanical ventilation and vasopressor use among PICU-admitted children with severe DSS.

## Acknowledgments

We are grateful to the patients and administrative staff for their support with this study.

## Author contributions

**Conceptualization:** Luan Vo Thanh, Thanh Nguyen Tat, Phuong Mai Ngo Thi.

**Data curation:** Luan Vo Thanh, Thanh Nguyen Tat, Bao Nguyen Trung, Dung Thuy Pham Thi, Thanh Hoai Mai Thi.

**Formal analysis:** Luan Vo Thanh, Thanh Nguyen Tat.

**Funding acquisition:** Thach Pham Ngoc, Tung Trinh Huu.

**Investigation:** Luan Vo Thanh, Thanh Nguyen Tat, Thach Pham Ngoc, Tung Trinh Huu.

**Methodology:** Luan Vo Thanh, Thanh Nguyen Tat, Thach Pham Ngoc, Tung Trinh Huu.

**Project administration:** Luan Vo Thanh, Thanh Nguyen Tat.

**Resources:** Luan Vo Thanh, Bao Nguyen Trung, Phuong Mai Ngo Thi, Dung Thuy Pham Thi, Thanh Hoai Mai Thi.

**Software:** Bao Nguyen Trung, Phuong Mai Ngo Thi, Dung Thuy Pham Thi, Thanh Hoai Mai Thi.

**Supervision:** Luan Vo Thanh.

**Validation:** Thanh Nguyen Tat.

**Visualization:** Thanh Nguyen Tat.

**Writing – original draft:** Luan Vo Thanh, Thanh Nguyen Tat.

**Writing – review & editing:** Luan Vo Thanh, Thanh Nguyen Tat, Bao Nguyen Trung, Phuong Mai Ngo Thi, Dung Thuy Pham Thi, Thanh Hoai Mai Thi, Thach Pham Ngoc, Tung Trinh Huu.
